# Halftone spatial frequency domain imaging enables kilohertz high-speed label-free non-contact quantitative mapping of optical properties for strongly turbid media

**DOI:** 10.1038/s41377-021-00681-9

**Published:** 2021-12-09

**Authors:** Yanyu Zhao, Bowen Song, Ming Wang, Yang Zhao, Yubo Fan

**Affiliations:** 1 Beijing Advanced Innovation Center for Biomedical Engineering, Key Laboratory for Biomechanics and Mechanobiology of Ministry of Education, School of Engineering Medicine, and with the School of Biological Science and Medical Engineering, Beihang University, 100191 Beijing, China; 2 Institute of Spacecraft Application System Engineering, China Academy of Space Technology, 100094 Beijing, China; 3 Beijing Institute of Spacecraft Engineering, 100094 Beijing, China

**Keywords:** Biophotonics, Imaging and sensing

## Abstract

The ability to quantify optical properties (i.e., absorption and scattering) of strongly turbid media has major implications on the characterization of biological tissues, fluid fields, and many others. However, there are few methods that can provide wide-field quantification of optical properties, and none is able to perform quantitative optical property imaging with high-speed (e.g., kilohertz) capabilities. Here we develop a new imaging modality termed halftone spatial frequency domain imaging (halftone-SFDI), which is approximately two orders of magnitude faster than the state-of-the-art, and provides kilohertz high-speed, label-free, non-contact, wide-field quantification for the optical properties of strongly turbid media. This method utilizes halftone binary patterned illumination to target the spatial frequency response of turbid media, which is then mapped to optical properties using model-based analysis. We validate the halftone-SFDI on an array of phantoms with a wide range of optical properties as well as in vivo human tissue. We demonstrate with an in vivo rat brain cortex imaging study, and show that halftone-SFDI can longitudinally monitor the absolute concentration as well as spatial distribution of functional chromophores in tissue. We also show that halftone-SFDI can spatially map dual-wavelength optical properties of a highly dynamic flow field at kilohertz speed. Together, these results highlight the potential of halftone-SFDI to enable new capabilities in fundamental research and translational studies including brain science and fluid dynamics.

## Introduction

The ability to quantify optical properties (i.e., absorption and scattering) of strongly turbid media has major impacts on the characterization of biological tissues, fluid fields, and many others^1–4^. With absorption spectra, the concentration of structural and functional tissue components (such as oxy-hemoglobin, deoxy-hemoglobin, water and lipids) can be easily calculated using Beer’s law^2,5^. Dynamics in the concentrations and spatial distribution of those components are hallmarks of many physiological and disease conditions including tissue oxygenation, metabolism, cardiovascular disease^6,7^, inflammation^8^, diabetes^9,10^, and several cancers^11^. Furthermore, while the concentration and spatial distribution of the combustion residues such as aqueous vapor can be obtained from optical property measurements, high-speed quantitative imaging of the turbid combustion flow is critical for the design of aeronautical and astronautical devices, such as airplane and rocket jet engines^12,13^. However, the task of quantitative imaging of optical properties for strongly turbid media is intrinsically challenging as photon scattering hinders direct measurement over length scales larger than the mean free path. Current imaging techniques such as photoacoustic imaging (PA) can probe absorbing contents in tissue such as hemoglobin^14,15^, but is unable to obtain quantitative absorption and scattering values^16,17^. Techniques such as hyperspectral imaging are more accessible^18^, but cannot reliably quantify the concentrations of specific tissue chromophores due to the confounding effects of optical absorption and scattering. Diffuse optical spectroscopy (DOS) can separate absorption from scattering in turbid media with carefully designed light modulation and detection. However, it is limited to point measurements, requires mechanical contact with the sample, and typically takes 10-20 min to acquire an image (i.e., on the order of 1e-3 Hz for imaging)^19,20^. Few current imaging technologies can quantify absorption and scattering properties of strongly turbid media in a wide-field non-contact format, and none have been shown to map quantitative optical properties with high-speed (e.g., kHz) capabilities. Here we address this need through a new optical imaging method that for the first time can quantify optical properties of strongly turbid media as well as functional chromophore concentrations in tissue in a high-speed, label-free, non-contact, and wide-field manner.

### Diffuse optics for the quantification of optical properties and chromophore concentrations in turbid media

The ability to quantify optical properties (i.e., absorption and scattering) of turbid media is intrinsically challenging due to the fact that optical absorption and scattering both contribute to the overall imaging contrast^5,21^. The inherent convolution of these physical effects also severely constrains the ability of most wide-field optical imaging techniques to quantify light-absorbing chromophores in the turbid media, such as oxy-hemoglobin, deoxy-hemoglobin, water, and lipids in tissue, and the amount of aqueous vapor in the combustion flow of airplane and rocket jet engines. Frequency-domain and time-domain diffuse optical techniques have been developed to isolate the effects of optical absorption and scattering using a combination of modulated illumination source and model-based analysis, typically utilizing specific solutions to the diffusion approximation of the Boltzmann transport equation, or from Monte Carlo simulations^22,23^. After the optical absorption is determined at multiple wavelengths, the major light-absorbing components in the turbid media can be quantified by solving a set of linear equations involving the known extinction spectra of each chromophore^5,24–26^.

Unfortunately, current diffuse optical imaging techniques are relatively slow for wide-field quantification of optical properties. Diffuse optical spectroscopy (DOS) is a representative temporal frequency domain technique which can isolate optical absorption and scattering by modulating the incident light at MHz frequency and collecting the amplitude and phase values of the reflected light^19,20^. However, it requires mechanical scanning to form an image of the sample, and each point measurement takes 15–30 s, leading to a measurement time of 10–20 min for a 20 × 20 pixel image (i.e., imaging speed on the order of 1e−3 Hz)^19,20^. To date, no diffuse optical techniques have been developed for kilohertz high-speed wide-field quantification of optical properties.

### Halftone spatial frequency domain imaging

Here, we develop a new optical technique, *halftone spatial frequency domain imaging*, or halftone-SFDI, for high-speed label-free non-contact wide-field quantification of optical properties in turbid media. Spatial frequency domain imaging (SFDI) is an emerging diffuse optical technology that recently gains significant attention^2,23,27–29^. It can quantify optical properties of turbid media in a label-free, non-contact manner, and its camera-based detection scheme makes it intrinsically wide-field. It projects spatially modulated sinusoidal light patterns of different phases onto the sample using a digital-micromirror-device (DMD) and collects the reflectance images. However, current SFDI technologies utilize a continuous-tone strategy and generate those sinusoidal patterns with 8-bit grayscale, which correspondingly has a limited maximum projection speed of 290 Hz determined by the DMD hardware (e.g., model V-7001, ViALUX). Consequently, while SFDI typically requires 5 projection patterns for the measurement of optical properties at a single wavelength, current SFDI technologies are severely limited for high-speed applications.

To address the bottleneck of measurement speed, we propose a halftone strategy to significantly increase the speed of SFDI by approximately two orders of magnitude, with no extra cost or modification on system hardware. In this work we first demonstrate the sinusoidal patterns generated by the halftone strategy for 1-bit DMD projection, which leads to a maximum projection rate of 23 kHz, approximately two orders of magnitude faster than that in current SFDI technologies. We also validate the proposed halftone-SFDI against conventional SFDI measurements through experiments on an array of optical phantoms with a wide range of optical properties as well as in vivo human tissue. We then demonstrate dynamic monitoring of wide-field optical properties and functional chromophore concentrations in the rat brain cortex with the proposed halftone-SFDI. Enabled by the proposed method, we also demonstrate kHz high-speed dual-wavelength monitoring of wide-field optical properties of a highly dynamic flow field. To the best of our knowledge, this is the first demonstration of label-free non-contact imaging of quantitative optical properties of strongly turbid media with a speed of kilohertz. We conclude with a discussion of basic science and engineering application areas in which halftone-SFDI may have a substantial impact.

## Results

### Halftone-SFDI principles and model-based data analysis

**Fig. 1:**
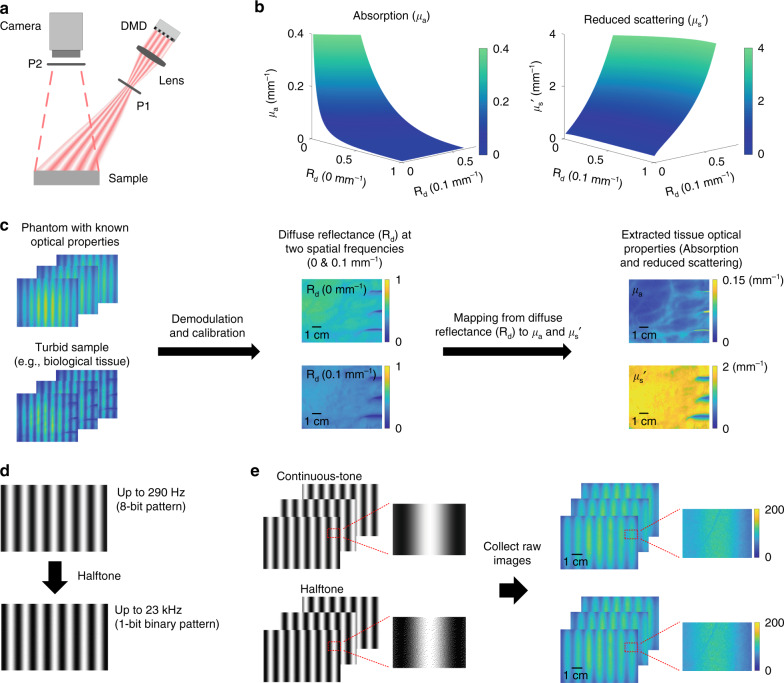
Halftone-SFDI principles and data flow. **a** Spatially modulated light patterns are generated by the DMD and projected onto the sample. Remitted light is collected by a camera. **b** A Monte Carlo based model is used to extract optical absorption (µ_a_) and reduced scattering (µ_s_′) from calibrated diffuse reflectance (*R*_d_) at two spatial frequencies, in this case 0 mm^−1^ and 0.1 mm^−1^. **c** Raw reflectance images are demodulated and calibrated to extract the spatial frequency response of the sample (i.e., diffuse reflectance). The Monte Carlo based inversion model is used to extract optical properties (absorption and reduced scattering) on a pixel-by-pixel basis. Here, an 8 × 6 cm region of human hand was imaged at 650 nm and the extracted optical absorption and reduced scattering maps are shown for the measured wavelength. **d** Conventional 8-bit continuous-tone illumination pattern is converted to halftone 1-bit pattern which increases the maximum projection rate from 290 Hz to 23 kHz, by approximately two orders of magnitude. **e** Raw images are collected for the same turbid sample region (i.e., human hand) with the conventional continuous tone and the proposed halftone illumination patterns, respectively. While the binary discontinuity is visible in the halftone patterns after zoomed-in, the corresponding collected images have identical appearance with the ones from continuous-tone patterns as a result of low-pass filtering effect of turbid media

Figure 1a shows the system diagram. A sequence of spatially modulated light patterns is generated by the DMD and projected onto the sample through a projection lens. There are five projection patterns for the measurement of each wavelength, including planar illumination, black image for dark measurement, and three patterns corresponding to 0°, 120°, and 240° phases of a specific spatial frequency. The corresponding reflectance images are then collected by the camera whose imaging rate is synchronized with the illumination. Cross-polarizers (P1 and P2 in the diagram) are placed after the projection lens and in front of the camera, respectively, to minimize specular reflection. The extraction of optical properties (i.e., absorption and reduced scattering) is conducted by direct mapping from the diffuse reflectance of two spatial frequencies using a pre-computed look-up-table (LUT) generated by Monte Carlo simulations of photon propagation in turbid media^30^, as shown in Fig. 1b. The Monte Carlo LUT maps diffuse reflectance values measured at the two spatial frequencies (e.g., 0 and 0.1 mm^−1^) to a unique combination of µ_a_ and µ_s_′^23,31^. The data flow of Monte Carlo model-based analysis is shown in Fig. 1c. First, a phantom with known optical properties and the turbid sample are respectively measured with selected spatial frequencies, in this case DC (0 mm^−1^) and meso-scale AC (0.1 mm^−1^) spatial frequencies. Although other combinations of spatial frequencies can also be used, it has been previously shown that this combination is effective for the extraction of optical properties (absorption and reduced scattering, denoted as µ_a_ and µ_s_′, respectively) in a wide range of tissues^32,33^. Then through demodulation and calibration, diffuse reflectance maps of the sample can be obtained for the selected spatial frequencies. The optical properties of the sample are finally calculated on a pixel-by-pixel basis using the pre-computed Monte Carlo LUT^30^. As shown in Fig. 1d, current SFDI technologies utilize continuous-tone 8-bit projection patterns on the DMD, which has a limited maximum projection rate of 290 Hz. In contrast, when converted to the proposed 1-bit binary halftone patterns, the maximum projection speed is increased to 23 kHz, which is improved by 80×, approximately two orders of magnitude faster than current SFDI technologies. Figure 1e further compares the continuous-tone and halftone patterns as well as the correspondingly collected images for the same region of a turbid sample (i.e., human tissue). Compared to the images collected with continuous-tone patterns, it can be seen that while the binary discontinuity is visible in the halftone projection patterns, the corresponding collected raw images have identical appearance without those discontinuities, which is due to the low-pass filtering effect of the turbid media and therefore allows the improvement of measurement speed by two orders of magnitude with those 1-bit halftone patterns.

### Phantom validation of halftone-SFDI

**Fig. 2:**
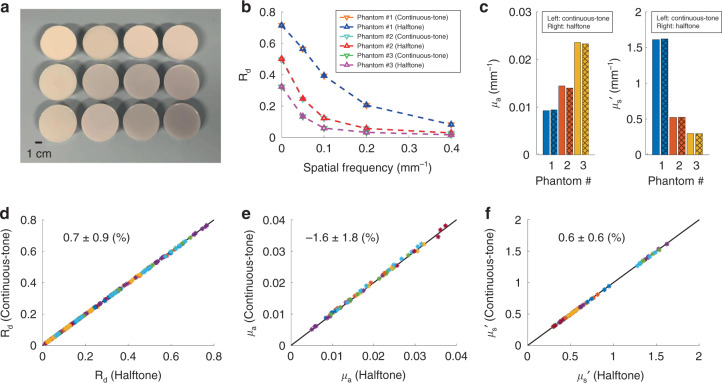
Validation of halftone-SFDI on an array of phantoms with a wide range of optical properties. **a** White light image of the fabricated array of 16 optical phantoms. **b** Comparison of diffuse reflectance at 650 nm of five spatial frequencies measured by the continuous-tone SFDI and the proposed halftone-SFDI for three representative phantoms. **c** Extracted optical properties of the three representative phantoms in (**b**). The hatch pattern was added to aid data interpretation. **d** Comparison of measured *R*_d_ values from 650 nm to 850 nm with 50 nm increments with five spatial frequencies for all the 16 phantoms. **e** Corresponding optical absorption values of the 16 phantoms at five wavelengths by the two competing methods, with a percent difference of −1.6 ± 1.8%. **f** Corresponding reduced scattering values of the 16 phantoms at five wavelengths by the two competing methods, with a percent difference of 0.6 ± 0.6%

The proposed halftone-SFDI is validated against established continuous-tone SFDI in a phantom study for the extraction of optical properties. A number of 16 optical phantoms were fabricated with a wide range of optical properties, with optical absorption in the range of 0.005—0.04 mm^−1^, and reduced scattering in the range of 0.3 – 2 mm^−1^. A white light image of those phantoms is shown in Fig. 2a. The diffuse reflectance of five spatial frequencies (0, 0.05, 0.1, 0.2, and 0.4 mm^−1^) was measured with halftone-SFDI and continuous-tone SFDI, respectively, at five wavelengths ranging from 650 nm to 850 nm with 50 nm increments. While a combination of two or more spatial frequencies can be used to extract optical properties, in this study the optical absorption and reduced scattering values were calculated with a spatial frequency combination of 0 and 0.1 mm^−1^ ^32^. The diffuse reflectance (R_d_) measured by the two methods at 650 nm of the five spatial frequencies is plotted in Fig. 2b for three representative phantoms (i.e., the first column of the phantom array in Fig. 2a). In Fig. 2b, the *R*_d_ values from the two methods are shown as downward and upward triangles, respectively, while the dashed lines are used to aid visualization. It can be seen that the diffuse reflectance values by the two methods overlap with each other. The extracted optical properties of those three phantoms are shown in Fig. 2c, where the extracted values by the two methods indicate good agreement. Furthermore, the diffuse reflectance measured by the two methods at those five spatial frequencies of all 16 phantoms is compared in Fig. 2d, with a percentage difference of 0.7 ± 0.9%. The percent difference is calculated using the values from the continuous-tone SFDI as reference. In addition, the extracted optical absorption values of the two methods for the 16 phantoms at five wavelengths are compared in Fig. 2e, with a percent difference of −1.6 ± 1.8%. The related noise level for optical absorption measurement was 2.5%. Additionally, the corresponding reduced scattering values are compared in Fig. 2f, with a percent difference of 0.6 ± 0.6%, and the related noise level was 1.3%. The noise levels were determined by repeated measurements on the same optical phantom. Specifically, a number of 10 repeated measurements were conducted for the same phantom using continuous-tone SFDI, and corresponding absorption and reduced scattering values were extracted for each repeated measurement. The noise level was calculated as the standard deviation of the 10 optical property values divided by its average. Those results demonstrate that the proposed halftone-SFDI gives nearly identical measurement values for optical properties compared to the established SFDI technology (i.e., continuous-tone SFDI), and the differences in measurement values are within the noise level.

### In vivo human tissue measurements

**Fig. 3:**
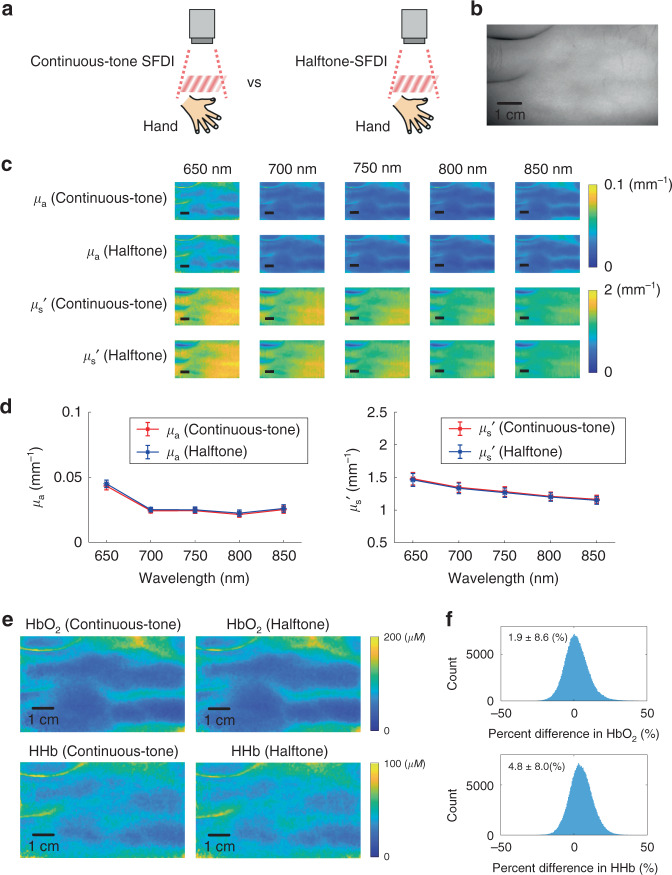
Comparison of halftone-SFDI with continuous-tone SFDI on in vivo human tissue measurements. **a** Illustration of measurements with the continuous-tone SFDI and halftone-SFDI methods. **b** Planar image of in vivo human hand tissue at 650 nm. **c** The tissue optical properties were measured with the two methods from 650 nm to 850 nm with 50 nm increments. **d** The average and standard deviation of the extracted optical properties are compared for the entire maps extracted by the two methods. **e** Functional chromophore maps were calculated from absorption spectra based on Beer’s law. **f** The differences in extracted hemoglobin concentrations were visualized and quantitatively compared

In addition to the phantom study, we further validate the proposed halftone-SFDI with the conventional SFDI for in vivo human tissue measurements, as shown in Fig. 3a. Figure 3b shows an image of the human hand measured under 650 nm with planar illumination. Both continuous-tone and halftone measurements were conducted using 0 and 0.1 mm^−1^ spatial frequencies with 650–850 nm wavelengths of 50 nm increments. The extracted optical absorption (µ_a_) and reduced scattering (µ_s_′) of the two methods are shown in Fig. 3c, where the extracted optical property maps appear visually identical. Those µ_a_ and µ_s_′ maps are then quantitatively compared in Fig. 3d. The average and standard deviation of the extracted values from the entire maps are shown in Fig. 3d, where the extracted values almost overlap with each other, and the average µ_a_ and µ_s_′ values of the two methods have percent differences of 3.0 ± 0.9% and −1.0 ± 0.3%, respectively, indicating good agreement. With the obtained optical absorption spectra, the functional chromophore concentrations were extracted based on Beer’s law. Figure 3e shows the extracted concentration maps for oxy-hemoglobin (HbO_2_) and deoxy-hemoglobin (HHb) using absorption measurements from the continuous-tone SFDI and the proposed halftone-SFDI, respectively. The hemoglobin maps from the two methods again appear visually identical. The histograms of percent difference in extracted hemoglobin concentrations are plotted in Fig. 3f and the percent differences are quantitatively compared for the entire concentration map. The percent differences for oxy-hemoglobin and deoxy-hemoglobin are 1.9 ± 8.6% and 4.8 ± 8.0%, respectively. In addition, the noise level for the extraction of chromophores was estimated with 10 repeated hand measurements and was calculated as the standard deviation of the 10 chromophore values divided by its average. The noise level was determined to be 4.2% for oxy-hemoglobin and 7.6% for deoxy-hemoglobin, respectively. Those results above indicate equivalence of the proposed halftone-SFDI with the conventional SFDI in terms of measurements in diffuse reflectance, optical properties, and chromophore concentrations.

### In vivo hemodynamics monitoring for rat brain cortex

**Fig. 4:**
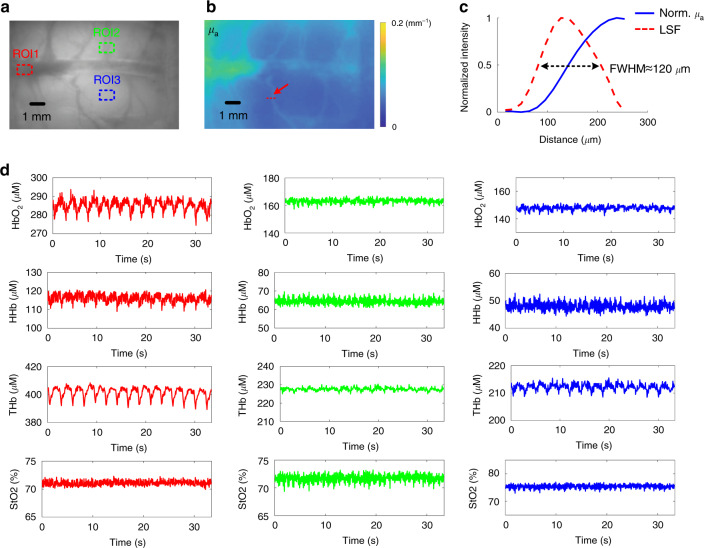
In vivo hemodynamics monitoring of rat brain cortex. **a** Planar image of the rat brain cortex at 685 nm. ROIs indicated by the dashed rectangles were selected on the middle cerebral vessel and cortex areas for visualization of the longitudinal hemodynamics. **b** Extracted optical absorption map at 685 nm. A tissue-vessel area was used to quantify spatial resolution in the absorption map, as indicated by the red dashed line. **c** The normalized line profile of absorption values is shown for the tissue-vessel area in (**b**) and used as edge spread function (ESF). The line spread function (LSF) was calculated by taking derivative of the ESF, and the spatial resolution, measured as the FWHM of the LSF, was 120 μm. **d** The functional chromophore concentrations and oxygenation level were monitored at 15 Hz for over 30 s. The time series of those physiological parameters is shown for the three representative ROIs, respectively

Small animals are widely used for preclinical studies, and the hemodynamics of their brain has major implications in brain science, neuroscience, and cognitive psychology^34–37^. As a proof-of-principle, we demonstrate quantitative hemodynamics monitoring of rat brain cortex with the proposed halftone-SFDI. The animal was under approximately 2% isoflurane anesthesia with a portion of the skull removed. The exposed brain cortex (11 × 7 mm) was imaged at a rate of 15 Hz using [685, 850] nm wavelengths and [0, 0.1] mm^−1^ spatial frequencies. Those two wavelengths were selected because they have been shown effective for the extraction of oxy- and deoxy-hemoglobin concentrations^38^. The 15 Hz speed was used as a result of available near-infrared camera in the lab. As will be demonstrated in the next section, kilohertz dual-wavelength monitoring can also be easily achieved with the proposed halftone-SFDI. The planar image of the rat brain cortex at 685 nm is shown in Fig. 4a, and the corresponding optical absorption map is shown in Fig. 4b. While the resolution of SFDI is dependent on optical properties, as indicated by the dashed red line in Fig. 4b, a tissue-vessel area was used to get a sense of the spatial resolution for the rat cortex imaging. The normalized line profile of optical absorption at 685 nm for the tissue-vessel area was plotted in Fig. 4c and used as the edge spread function (ESF). The corresponding line spread function (LSF) was obtained by taking derivative of the ESF, and the full width at half maximum (FWHM) was calculated and shown in Fig. 4c, demonstrating a spatial resolution of 120 μm. In order to demonstrate the longitudinal monitoring of hemodynamics, three ROIs were selected in the brain area, including the middle cerebral vessel and the cortex areas, shown as red, green, and blue dashed rectangles respectively in Fig. 4a. With measured absorption at the two wavelengths, the functional chromophore concentrations and oxygenation level were calculated using Beer’s law, including oxy-hemoglobin concentration, deoxy-hemoglobin concentration, total hemoglobin concentration (THb), and oxygenation (StO2). The corresponding time series of the average values for the three ROIs are shown in Fig. 4d. The first column in Fig. 4d shows the time series of functional chromophore concentrations and oxygenation level of the middle cerebral vein, where the periodic physiological signals are clearly visible. The time series of functional chromophore concentrations and oxygenation level of the cortex areas are shown in the second and third columns in Fig. 4d. As expected, the periodic signals from the cortex areas are less apparent compared to those measured from the vessel area.

### High-speed monitoring of optical properties for highly dynamic flow field

**Fig. 5:**
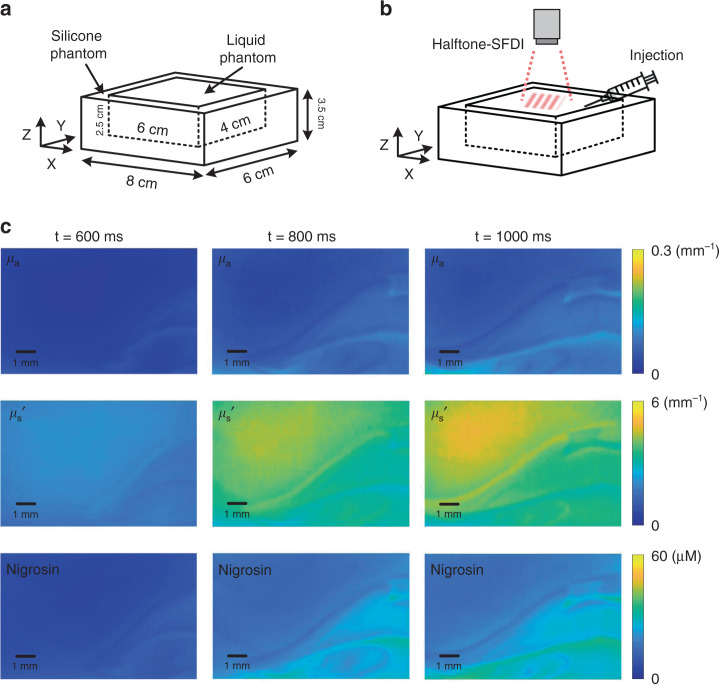
High-speed quantitative monitoring of highly dynamic flow field. **a**. A 8 × 6 × 3.5 cm silicone phantom was fabricated with a 6 × 4 × 2.5 cm well as container. The well was initially filled with liquid phantom (60 ml 1% intralipid) before monitoring. **b**. The halftone-SFDI was used to monitor the center of the liquid phantom with a 1 × 0.7 cm field of view. The monitoring was conducted at 1000 Hz with 470 nm and 625 nm wavelengths. A dynamic flow with absorption and scattering contrasts was induced by injection of a solution made with nigrosin and 20% intralipid. **c** The extracted absorption and reduced scattering maps, as well as chromophore maps at three representative time points are demonstrated for the quantitative monitoring of the flow field. A complete video showing the process is included as supplementary file. (The video was compressed and converted from original frames to meet the upload requirement)

High-speed quantitative monitoring of flow field has major implications for areas including fluid dynamics and combustion dynamics. However, current high-speed methods for quantification of flow field optical properties (i.e., absorption and scattering) are limited by single point measurements^12,13^. As a proof-of-principle, here we demonstrate high-speed wide-field quantitative monitoring of optical properties for a highly dynamic flow field using the proposed halftone-SFDI with an unprecedented speed of 1000 Hz. Figure 5a shows the geometry of the measurement scene. An 8 × 6 × 3.5 cm silicone phantom with a 6 × 4 × 2.5 cm well was fabricated as a container. The well was initially filled with liquid phantom made of 60 ml 1% intralipid. As shown in Fig. 5b, halftone-SFDI was used to continuously monitor the center of the liquid phantom with a 1 × 0.7 cm field of view. The halftone-SFDI monitoring was conducted at 1000 Hz with dual-wavelengths (i.e., 470 nm and 625 nm) and [0, 0.1] mm^−1^ spatial frequencies. The wavelengths were selected to accommodate the spectral response of the available high-speed camera which is sensitive to visible wavelengths. The spatial frequencies were selected since they have been previously shown effective for the extraction of a wide range of optical properties^32^. While a number of five illumination patterns was used for each wavelength, the DMD, LED light source, and the camera were synchronized and operated at 10,000 Hz. A highly dynamic flow with absorption and scattering contrasts was induced by injection of 0.6 ml solution made with nigrosin and 20% intralipid. Figure 5c shows the extracted optical absorption and reduced scattering images of 470 nm, as well as chromophore (i.e., nigrosin) concentration maps at three representative time points during the monitoring, where the evolution of the flow field is quantitatively mapped and clearly visualized. The high-speed monitoring was conducted for a total of 5 s. The corresponding complete video showing the entire sequence of extracted optical properties and chromophore maps is included as supplementary file.

## Discussion

We have developed and validated halftone-SFDI as a new method for high-speed, label-free, and non-contact imaging of optical properties of turbid media. This is enabled through the use of binary sinusoidal projection patterns (i.e., halftone patterns) which is able to take full advantage of the high-modulation rate of the DMD. The spatially modulated illumination in combination with a light-transport model allows for the separation of optical absorption and scattering effects in the turbid media. The extracted optical absorption at multiple wavelengths further enables quantitative mapping of light-absorbing components in the media, such as functional chromophore concentrations in tissue. In comparison to the conventional SFDI technologies, the proposed halftone-SFDI is approximately two orders of magnitude faster. Additionally, through validation on an array of phantoms with a wide range of optical properties, we demonstrated that the proposed halftone-SFDI gave equivalent measurement values on diffuse reflectance as well as optical absorption and reduced scattering for different spatial frequencies and wavelengths. In addition to the phantom study, we further validated the proposed halftone-SFDI on in vivo human tissue measurements in terms of optical properties and chromophore concentrations. We then demonstrated the halftone-SFDI for label-free in vivo hemodynamics monitoring of rat brain cortex. Finally, to highlight the high-speed wide-field monitoring capability enabled by the proposed method, we demonstrated monitoring of a highly dynamic flow field at an unprecedented speed of 1000 Hz with quantitative mapping of dual-wavelength optical properties as well as chromophore concentration.

Halftone-SFDI enjoys a number of advantages over methods currently used for probing turbid media. Compared to popular diffuse optical spectroscopy (DOS) techniques that can also quantify optical absorption and scattering values, halftone-SFDI can quantitatively map optical absorption and scattering in a wide-field and non-contact manner, while DOS can only give point measurements and requires sample contact. Diffuse optical spectroscopic imaging (DOSI) is an extension of DOS which can give maps of optical properties. However, the spatial resolution of DOSI is approximately 1 cm^26^, and the measurement requires point-by-point scanning over the sample area which takes 10–20 min to acquire a 20 × 20 pixel image (i.e., imaging speed on the order of 1e−3 Hz)^19,20^. In contrast, the proposed halftone-SFDI achieved a spatial resolution of 120 μm and imaging speed of kilohertz. In addition, while photoacoustic imaging (PA) can also probe absorbing contents in strongly turbid media^14,15^, it cannot quantitatively separate absorption from scattering^16,17^. It is therefore challenging for PA to measure absolute concentrations of different light-absorbing contents in turbid media. Furthermore, compared to conventional SFDI technologies, the proposed halftone-SFDI is 80× faster, an improvement of approximately two orders of magnitude.

This work has several important implications for scientific research and engineering applications. For example, mapping brain cortex functions is of fundamental significance for brain science, neuroscience, and cognitive psychology^1,34–37^. Current methods such as two-photon imaging and fluorescence imaging are limited by a relatively small field of view or requires exogenous contrast agents. The proposed halftone-SFDI can provide both larger field of view (e.g., multiple centimeters) and label-free quantitative functional imaging. The field of view can be further extended to multiple meters with sufficient illumination power. While we demonstrated quantitative monitoring of rat brain cortex in this study, it is noted that the halftone-SFDI can be easily implemented for human brain imaging with potential applications for high-speed wide-field hemodynamics monitoring in brain surgery. Furthermore, in terms of temporal resolution, the halftone-SFDI achieves kilohertz monitoring which is similar to that of EEG methods, while the latter is known to have limited spatial resolution and requires mechanical contact^39,40^. In addition, compared with single-snapshot SFDI methods^41^, the proposed halftone-SFDI would be able to capture highly dynamic physiological phenomena in the brain such as hemodynamic response triggered by electric stimulation^42^. These hemodynamic changes are typically coupled with neural activities and are important in neuroscience studies^36^. Furthermore, while demodulation with small field of view (e.g., 5–10 mm field of view in neuroscience studies with small animals) would be challenging for single-snapshot methods, the demodulation of halftone-SFDI is not limited by the size of field of view. Therefore, the halftone-SFDI can be a powerful tool for research related to the brain for both preclinical and clinical applications. Particularly, it would be interesting to combine halftone-SFDI with EEG to study the entanglement between neural activities and hemodynamics of the whole cortex with multi-kHz speed. In addition, we have demonstrated kilohertz mapping of quantitative optical properties and chromophore concentration in highly dynamic flow field, which suggests a wide range of applications related to fluid dynamics. For example, the amount of aqueous vapor in the turbid combustion flow is closely related to the energy efficiency of airplane and rocket jet engines. Current technology such as tunable diode laser absorption spectroscopy (TDLAS) is limited to point measurements^12,13^. In contrast, with shortwave-infrared wavelengths, the halftone-SFDI can provide kHz wide-field quantification of water content in the turbid combustion flow^2^, which could have a substantial impact on the design and optimization of those engines and dramatically reduce related energy cost.

Finally, it is important to note the potential maximum speed of halftone-SFDI for the extraction of optical properties as well as chromophore concentrations. Taking monitoring of the rat brain cortex and the highly dynamic flow field as an example, in the experiments we followed the vanilla version of SFDI and used a number of five different projection patterns (i.e., planar image, dark image, and three images at 0°, 120°, and 240° phases respectively of a specific spatial frequency) to measure optical properties at each wavelength. On the other hand, it has been previously shown that the planar image can also be obtained by taking average of the three-phase images. In addition, while the dark image is subtracted from the planar image in post-processing to account for ambient light, such subtraction may not be necessary for measurements in a dark environment or can be taken only once assuming a stable ambient illumination. Therefore, a minimum of only three projection patterns is required for optical property measurement of each wavelength. Given the DMD’s maximum projection rate of 23 kHz for binary patterns, it leads to a maximum speed of 7.6 kHz for halftone-SFDI to map optical properties in turbid media at a single wavelength. Furthermore, since it requires absorption values of two wavelengths to calculate the concentration of functional chromophores in tissue (i.e., oxy-hemoglobin and deoxy-hemoglobin), the halftone-SFDI can achieve a maximum speed of 3.8 kHz for mapping absolute concentrations of those chromophores as well as tissue oxygenation. Additionally, it is also important to note the potential limitation of halftone-SFDI. Despite the fact that halftone-SFDI is able to measure optical properties at multi-kilohertz speed, the acquired data need to be postprocessed. For example, in the flow field monitoring experiment, the demodulation and LUT optical property extraction took 1.2 ms and 0.8 s, respectively, for each wavelength. This makes the use of halftone-SFDI challenging for real-time kilohertz mapping of optical properties, although the development of ultrafast deep learning processing algorithms combined with advanced hardware such as GPU and FPGA might overcome this limitation^27,28,31,43^.

In summary, we have developed halftone-SFDI, a new label-free non-contact optical imaging modality that can spatially map quantitative optical absorption and scattering properties with a maximum speed of 7.6 kHz in strongly turbid media, and can map absolute functional chromophore concentrations in tissue with a maximum speed of 3.8 kHz. This modality fills an important gap left by other imaging modalities through its capability to measure optical properties and functional chromophore concentrations in a wide-field manner with multi-kilohertz speed, and without the use of exogenous agents and mechanical contact of the sample. We have demonstrated applications including in vivo monitoring of rat brain cortex and high-speed monitoring of highly dynamic flow field. Based on our findings, halftone-SFDI has the potential for significant impact in both fundamental research and translational applications.

## Materials and methods

### Imaging system setup

The main components of the SFDI imaging system were the multi-wavelength light source, DMD, and the camera. Specifically, a 1000 W halogen lamp light source (HL1000, NBeT, Beijing, China) was coupled to a monochromator (HGISW151, Yunke Instrument, Shandong, China) to provide illumination of different wavelengths. The spatial modulation was conducted using a DMD (V-650L, ViALUX, Saxony, Germany). A FLIR camera (BFS-U3-04S2M-CS, FLIR, Oregon, United States) was used to collect images. The same hardware was used for the continuous-tone SFDI and the halftone-SFDI. In terms of software programs, the software from manufacturers were used to control corresponding hardware components, and those hardware components were set to external triggering mode. An Arduino board was used to output TTL signals to synchronize the light source, DMD, and the camera. In terms of programming languages, the Arduino programming language, MATLAB, and C++ were used to program and control the imaging system components.

In addition, an achromatic lens with 75 mm focal length and 50.8 mm diameter (GLH-31, Heng Yang Guang Xue, Shenzhen, China) was used as the projection lens. The camera lens had a 35 mm focal length (CW-FM3517-10MP, CW Video, Shenzhen, China). The imaging field of view was 7 × 4 cm, and the working distance was 30 cm (the field of view can be adjusted to accommodate different measurement scenarios by changing the distance between the projection lens and the DMD). The data processing was conducted using MATLAB (MathWorks Inc., Natick, Massachusetts, USA) on a desktop computer with an Intel i9-9900K 3.6 GHz CPU and 64 GB RAM.

### Demodulation and calibration for spatial frequencies

The demodulation for a specific spatial frequency is conducted with raw images of the three phases using $I = \frac{{\sqrt 2 }}{3}\sqrt {\left( {(I_1 + I_2)^2 + (I_2 + I_3)^2 + (I_3 + I_1)^2} \right)}$, where *I* is the intensity of the demodulated image, and *I*_1_, *I*_2_, and *I*_3_ represent the collected raw images of the three phases, respectively^44^. The demodulation extracts the response of the sample (e.g., turbid media) for a specific spatial frequency. After demodulation, calibration is conducted to remove instrument response to get the diffuse reflectance of the sample at each spatial frequency *f*_*x*_. The demodulated images of the sample and the calibration phantom at spatial frequency *f*_*x*_ are denoted as *I*_sample_ (*f*_*x*_) and *I*_phantom_ (*f*_*x*_), respectively. The diffuse reflectance image of the sample, denoted as *R*_d_sample_ (*f*_*x*_), is calculated using Eq. 1. The diffuse reflectance of the calibration phantom (i.e., *R*_d_phantom_ (*f*_*x*_)) is obtained from the established Monte Carlo model, which maps the known optical properties to diffuse reflectance values at different spatial frequencies.

1 $$R_{{\rm{d}}\_{\rm{sample}}}(f_x) = \frac{{I_{{\rm{sample}}}(f_x)}}{{I_{{\rm{phantom}}}(f_x)}} \ast R_{{\rm{d}}\_{\rm{phantom}}}(f_x)$$

### Halftone sinusoidal projection patterns

The conversion from continuous-tone to halftone patterns has been a longstanding topic in digital printing^45–47^. Error diffusion and iterative search are two commonly used methods to generate halftone patterns^45,46^. The error diffusion method utilizes neighborhood operation and thresholding whereas the term “error diffusion” refers to the process of diffusing the quantization error along the path of the image scan. On the other hand, the iterative methods such as direct binary search tries to minimize the difference between the halftone pattern and the original continuous-tone pattern through iterative search, which typically requires time-consuming optimizations to reach convergence. Due to simplicity and ease of implementation, in this work we used the error diffusion method to generate halftone sinusoidal projection patterns of intended spatial frequencies.

### Optical phantom fabrication and imaging

The calibration phantom used in experiments was made with 10% intralipid, whose optical properties can be found in literature^48,49^. In the phantom validation study, a total of 16 phantoms with a wide range of optical absorption and scattering properties were fabricated using nigrosin (N814749-100g, Macklin, Shanghai, China), titanium dioxide (TiO_2_) (PL975541-500g, Cool Chemistry, Beijing, China), silicone base and its curing agent (#906, Chunlan, Guangdong, China). Briefly, the amount of silicone base and curing agent were kept the same for different phantoms. The absorption property was adjusted by varying the amount of nigrosin, and the scattering property was adjusted by varying the amount of TiO_2_. In the flow field monitoring study, the initial liquid sample was made from 1% intralipid with a volume of 60 ml. A 0.6 ml solution was used for the injection, made from a mixture of 0.1 ml 0.4 g/L nigrosin solution and 0.5 ml 20% intralipid (Fresenius Kabi SSPC, Jiangsu, China). The imaging was conducted using the continuous-tone SFDI and the proposed halftone-SFDI, respectively. A wide range of wavelengths from 650 nm to 850 nm with 50 nm increments was measured for each phantom using both imaging methods, where a spatial frequency combination of [0, 0.05, 0.1, 0.2, 0.4] mm^−1^ was used for each wavelength. The optical properties were calculated using 0 and 0.1 mm^−1^ spatial frequencies, which has been previously shown effective for extracting optical properties^32^. The white Monte Carlo model was used in processing to map between diffuse reflectance values and optical properties^30^. In terms of hardware, a halogen lamp light source (HL1000, NBeT, Beijing, China) was coupled to a monochromator to provide illumination of different wavelengths. The spatial modulation was conducted using a DMD (V-650L, ViALUX, Saxony, Germany). A FLIR camera (BFS-U3-04S2M-CS, FLIR, Oregon, United States) was used to collect images.

### In vivo human tissue imaging

The human tissue imaging was conducted on the back of hand of a healthy volunteer. A 10% intralipid phantom was used for calibration, whose optical properties can be found in literature^48,49^. The measurement was conducted from 650 to 850 nm wavelengths in 50 nm increments, with 0 and 0.1 mm^−1^ spatial frequencies, using the same hardware as in the phantom study. The optical properties were extracted using the LUT generated from the Monte Carlo model. The chromophore concentrations were extracted using Beer’s law with absorption spectra measured at the five wavelengths. The experimental procedures were reviewed and approved by the Beihang University Biological and Medical Ethics Committee.

### In vivo monitoring of rat brain cortex

Sprague Dawley rat (220 g, female, 8 weeks old; Vital River Laboratories, China) was used in the experiment. The skull was removed to expose the brain cortex (11 × 7 mm). The animal was under anesthesia during the procedure with approximately 2% isoflurane anesthesia. All animal procedures were reviewed and approved by the Beihang University Biological and Medical Ethics Committee. The imaging was conducted using 0 and 0.1 mm^−1^ spatial frequencies with 685 nm and 850 nm LEDs. The chromophore concentrations were extracted using Beer’s law with optical absorption measured at the two wavelengths. The spatial modulation of the light patterns was conducted using a DMD (V-650L, ViALUX, Saxony, Germany). A FLIR camera (BFS-U3-04S2M-CS, FLIR, Oregon, United States) was used to collect images. The DMD, LED light source, and the FLIR camera were synchronized and operated at 150 Hz for longitudinal measurements.

### High-speed monitoring of optical properties of highly dynamic flow field

A 8 × 6 × 3.5 cm silicone phantom was fabricated to serve as a container with a 6 × 4 × 2.5 cm well in it. A 60 ml liquid phantom made of 1% intralipid was initially filled in the well. The highly dynamic flow field with optical absorption and scattering contrasts was induced by injection of a 0.6 ml solution to the liquid phantom. The injected solution was made with 0.1 ml 0.4 g/L nigrosin solution and 0.5 ml 20% intralipid. The nigrosin is a type of molecule with strong light absorption, and the 20% intralipid is highly scattering compared to the 1% intralipid base. The monitoring was conducted at 1000 Hz speed using 0 and 0.1 mm^−1^ spatial frequencies with 470 nm and 625 nm wavelengths. A DMD (V-650L, ViALUX, Saxony, Germany) was used to spatially modulate the illumination pattern. A high-speed camera (i-SPEED 3, iX-Cameras, United Kingdom) was used for image collection. The imaging lens had a 100 mm focal length (atx-i 100 mm F2.8 FF Macro, Tokina, Tokyo, Japan), and the working distance was approximately 10 cm. The DMD, LED light source, and the camera were synchronized and operated at 10,000 Hz for the high-speed halftone-SFDI measurements.

## Supplementary information

High-speed quantitative monitoring of highly dynamic flow field

## Data Availability

All the relevant data supporting the findings of this study are available within the paper, its Supplementary information, and from the corresponding author upon reasonable request.

## Code availability

All the relevant scripts supporting this study are available from the corresponding author upon reasonable request.
